# Inflammation and Oxidative Stress: Potential Targets for Improving Prognosis After Subarachnoid Hemorrhage

**DOI:** 10.3389/fncel.2021.739506

**Published:** 2021-09-24

**Authors:** Fan Wu, Zongchi Liu, Ganglei Li, Lihui Zhou, Kaiyuan Huang, Zhanxiong Wu, Renya Zhan, Jian Shen

**Affiliations:** ^1^First Affiliated Hospital, School of Medicine, Zhejiang University, Hangzhou, China; ^2^Department of Neurosurgery, First Affiliated Hospital, College of Medicine, Zhejiang University, Hangzhou, China; ^3^College of Electronics and Information, Hangzhou Dianzi University, Hangzhou, China

**Keywords:** subarachnoid hemorrhage, poor prognosis, delayed ischemic neurological deficit, inflammation, oxidative stress, anti-inflammatory, antioxidant

## Abstract

Subarachnoid hemorrhage (SAH) has a high mortality rate and causes long-term disability in many patients, often associated with cognitive impairment. However, the pathogenesis of delayed brain dysfunction after SAH is not fully understood. A growing body of evidence suggests that neuroinflammation and oxidative stress play a negative role in neurofunctional deficits. Red blood cells and hemoglobin, immune cells, proinflammatory cytokines, and peroxidases are directly or indirectly involved in the regulation of neuroinflammation and oxidative stress in the central nervous system after SAH. This review explores the role of various cellular and acellular components in secondary inflammation and oxidative stress after SAH, and aims to provide new ideas for clinical treatment to improve the prognosis of SAH.

## Introduction

Globally, six to nine in 100,000 people seek medical attention for subarachnoid hemorrhage (SAH) annually. More than 80% of SAHs are caused by ruptured intracranial aneurysms, with a mortality rate of 35% (Neifert et al., 2021). During aneurysmal SAH, increased intracranial pressure causes a sharp decrease in cerebral perfusion pressure that can lead to acute cerebral ischemia (CI) and loss of consciousness. Although SAH accounts for only 5% of all strokes, it imposes a significant health burden on society due to its young age of onset. Those who survive the initial bleeding often develop severe disability, with cognitive impairment, known as delayed ischemic neurological deficit (DIND) (Macdonald, 2014). Currently, poor long-term prognosis is attributed to delayed CI (DCI) in most cases. From day 5 to 14 after SAH, patients are at increased risk for DCI, which may be manifested as headache, confusion, focal neurological impairment, or decreased levels of consciousness (Østergaard et al., 2013).

Historically, spasm of the large arteries has been considered the only explanation for DCI. At present, angiography is still the gold standard for detecting vasospasm. However, even though up to 70% of SAH patients develop angiographic vasoconstriction, only about 50% develop DCI (Francoeur and Mayer, 2016). Nimodipine may reduce the incidence of DCI and the risk of poor prognosis after SAH by preventing and reducing vasospasm through muscle wall relaxation. Even though angiographic vasospasm has been successfully alleviated, prognosis has not improved (Etminan et al., 2011).

In recent years, it has been shown that factors other than vasospasm are involved in the pathophysiology of DCI, including microcirculation contraction, microthrombosis, cortical diffusion ischemia, and delayed apoptosis (Macdonald, 2014). Neuroinflammation and oxidative damage after SAH link these factors together.

**FIGURE 1:**
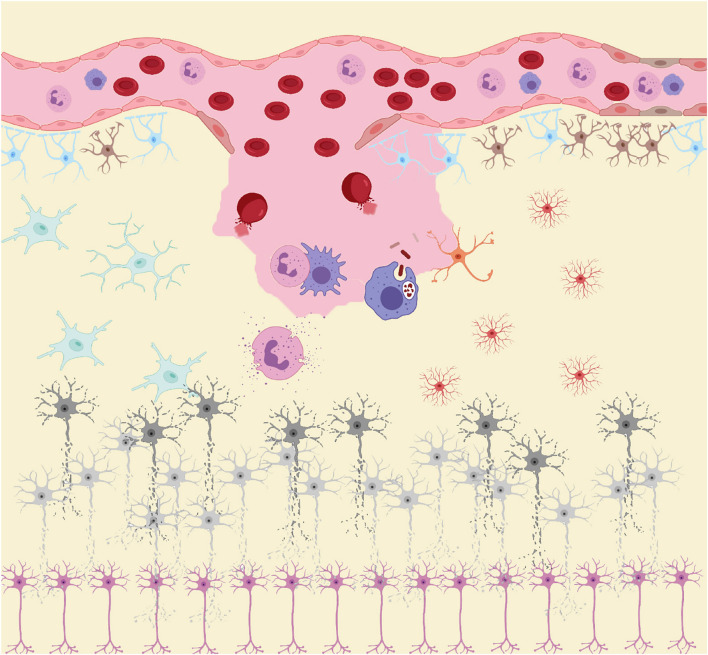
After subarachnoid hemorrhage (SAH), blood components enter the subarachnoid space. RBC rupture hemoglobin and its metabolites, together with other damage-associated molecular patterns (DAMPs), act as inducers of the secondary inflammatory response after SAH to activate the innate immune cells (microglia and astrocytes) in central nervous system (CNS). Subsequently, immune cells such as neutrophils and macrophages in the peripheral circulation infiltrate into the injured site under the action of chemokine recruitment. These peripheral immune cells, together with innate immune cells in CNS, act as the carriers of secondary inflammation after SAH, releasing large amounts of pro-inflammatory cytokines and peroxides causing damage to neurons. Under the influence of these inflammatory products and peroxides, neurons gradually appear cell dysfunction and even apoptosis.

Similar to the biphasic course of SAH (early bleeding and late ischemia), the inflammatory response in the pathological course is also biphasic (Lucke-Wold et al., 2016). In the acute stage, the main manifestation is the local inflammatory response caused by blood components entering the subarachnoid space and triggering downstream inflammatory cascades. Subsequently, in the subacute and chronic stages, while central resident immune cells are activated, a large number of peripheral inflammatory cells enter the subarachnoid space under the chemotactic influence of inflammatory cytokines (Figure 1; Zhou et al., 2014). As the final effector of the inflammatory response, inflammatory cells secrete a variety of inflammatory cytokines. Inflammatory cells and cytokines play an important role in the process of neurodegenerative and neurobehavioral disorders (Healy et al., 2020). Thus, inflammation plays a central role in the development of post-SAH complications. The release of oxyhemoglobin, mitochondrial dysfunction, and overexpression of peroxidase lead to excess oxidative products that exceed the body’s antioxidant capacity, leading to destruction of the blood–brain barrier (BBB), loss of neurons, glial hyperplasia, and permanent neurological impairment (Hu et al., 2016). While inflammation induces oxidative stress, oxidative stress can also induce an inflammatory response. The two complement each other and contribute to poor prognosis after SAH.

## Onset of Inflammation and Oxidative Stress

In the acute phase of SAH, the main manifestation is activation of local inflammation at the site of injury. Substances released from damaged cells and blood components enter the subarachnoid space as damage-associated molecular patterns (DAMPs) that act by initiating inflammation (Chaudhry et al., 2018). An increasing number of DAMPs has been identified, including high mobility group box (HMGB)1, heat shock protein (HSP), S100 protein, hemoglobin and its derivatives, mitochondrial DNA, IL-1α, IL-33, and extracellular matrix.

**FIGURE 2:**
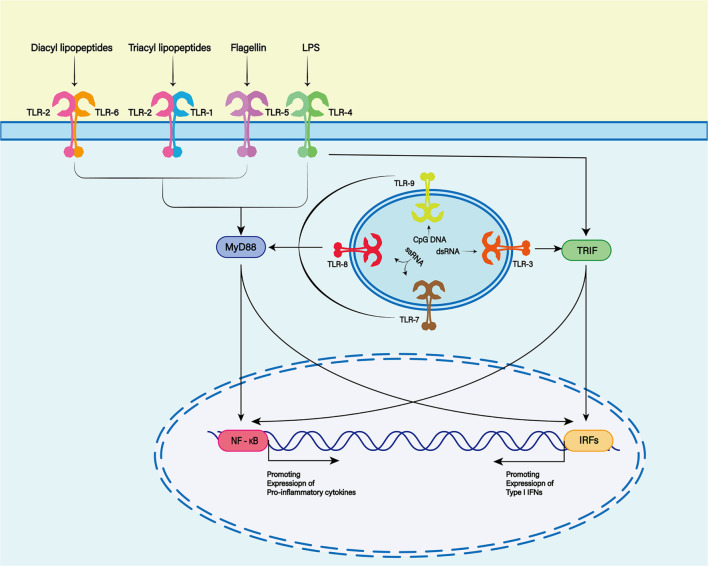
The Toll-like receptor (TLR) family plays a key role in recognizing antigens produced by microorganisms. To date, 13 TLR family members have been discovered. TLR1, 2, 4, 5, and 6 were expressed on the cell surface, while TLR3, 7, 8, and 9 were expressed on the endosome membrane. Toll-like receptors are membrane receptors composed of extracellular domains, single transmembrane helical domains and intracellular signaling domains, which can bind to different ligands (TLR2 and TLR1 or TLR6 complexes recognize lipoproteins or lipopeptides, TLR3 recognizes double-stranded RNA, TLR4 recognizes lipopolysaccharides (LPS), TLR5 recognizes bacterial flagellins, TLR7 or TLR8 recognizes single-stranded RNA, and TLR9 recognizes CpG rich in hypomethylated DNA). When the TLR binds to the respective ligands, different downstream signals can be activated to produce different biological effects.

Damage-associated molecular patterns are primarily recognized by pattern recognition receptors (PRRs), such as Toll-like receptors (TLRs), cytoplasmic NOD-like receptors (NLRs) and non-PRRs (such as CD44, integrin, and CD91 receptor) (Zindel and Kubes, 2020). The TLR family is one of the most characteristic PRR families, and is widely expressed in the membranes of glial cells such as microglia, astrocytes and oligodendrocytes in the central nervous system (CNS) (Pascual et al., 2021; Figure 2). TLR-4 plays a major role in the inflammatory response after SAH (Okada and Suzuki, 2017). TLR4 is an important member of the TLR family in PRRs and can be activated by hemoglobin and its derivatives (Leitner et al., 2019).

TLR4-dependent proinflammatory cytokines trigger an inflammatory response similar to that induced by lipopolysaccharide (Grylls et al., 2021). With the help of myeloid differentiation factor (MD)-2 and CD14, TLR4 can interact with two different junction proteins: Myeloid differentiation primary-response protein 88 (MyD88) and Toll receptor associated activator of interferon (TRIF) (Okada and Suzuki, 2017).

In the MyD88-dependent nuclear factor (NF)-κB signaling pathway, TIR domain containing adaptor protein/MyD88 adaptor-like (TIRAP/MAL) is required to bridge TLR and MyD88 when DAMPs bind to TLR4. MyD88 then interacts with interleukin-1 receptor (IL-1R)-associated kinase (IRAK)-4, which activates other members of the IRAK family, including IRAK-1 and IRAK-2. Activated IRAKs interact with tumor necrosis factor receptor (TNFR)-associated factor (TRAF)6 (Leitner et al., 2019). TRAF6 is associated with transforming factor-β-activated kinase (TAK)1, which leads to activation of the NF-κB signaling pathway and increased transcription and expression of proinflammatory cytokines (Karimy et al., 2020). In the TRIF-dependent signaling pathway, TLR4 requires TRIF-related adaptor molecule (TRAM) to activate TRIF. Subsequently, activated TRIF induces activation of the NF-κB signaling pathway by recruiting and activating TRAF6 or receptor-interacting serine/threonine protein kinase (RIPK)1 (Ciesielska et al., 2021).

Although the two different connective proteins act in different ways, both ultimately activate the NF-κB signaling pathway and trigger further inflammatory cascades (Takeuchi and Akira, 2010).

## Toxicity of Hemoglobin

Hemoglobin and its metabolites form a toxic cascade during early brain injury after SAH that is thought to play a key role in the development of delayed brain injury (Brathwaite and Macdonald, 2014). The neurotoxicity of hemoglobin is indisputable, and neurons seem to be more susceptible than glial cells (Li et al., 2012). The toxicity of hemoglobin is multifactorial, but is mediated mainly by four factors: inflammation, oxidation, nitric oxide (NO) depletion, and edema (Robicsek et al., 2020).

### Hemoglobin and Its Products Acting as DAMPs

Hemoglobin and hemoglobin-derived products are the most important DAMPs released by ruptured red blood cells (RBCs) in the subarachnoid space and are involved in the inflammatory activation of SAH (Bozza and Jeney, 2020). Hemoglobin and its products, such as methemoglobin, heme, heme chloride, and oxygenated hemoglobin, bind to the TLR-4 receptor as DAMPs. Methemoglobin is water soluble, which can lead to extensive TLR-4 activation with cerebrospinal fluid (CSF) circulation. In addition, heme stimulates the formation of more neutrophil extracellular traps (NETs) while activating TLR-4.

### Hemoglobin as a Source of Peroxide

Hemoglobin consists of four globin chains tightly bound to the heme group. Oxyhemoglobin and its metabolites are considered to be the main sources of reactive oxygen species (ROS) in the pathophysiological process of SAH (Hugelshofer et al., 2018). After hemolysis, tetrameric hemoglobin is released from RBCs and degrades gradually, producing toxic intermediates (Stokum et al., 2021). Among them, heme is considered more toxic than hemoglobin (Bulters et al., 2018). In the ferrous (Fe^2+^) and trivalent (Fe^3+^) states, heme can react with hydrogen peroxide to generate hydroxyl radicals through the Fenton reaction, which can damage lipid membranes and lead to the production of lipid ROS, cell dysfunction and even ferroptosis (Marnett et al., 2003; Yang and Stockwell, 2016).

These hemoglobin-derived products induce toxicity by producing ROS that can cause oxidation of cell lipids, proteins and DNA, leading to programmed cell death (Sinha et al., 2013). ROS can further activate TLR/NF-κB/MAPK, KEAP1–NRF2–ARE, eicosanoid and other signaling pathways as well as NLRP3 inflammasomes to mediate inflammatory responses (Reczek and Chandel, 2015). Oxyhemoglobin has been shown to induce cerebral artery contraction after SAH by inhibiting voltage-dependent K^+^ channels in cerebral arteries and inducing R-type Ca^2+^ channel expression in cerebral arteries (Ishiguro et al., 2006; Link et al., 2008).

### NO Depletion

As an important endogenous vasodilator, NO can be produced by endothelial cells, neurons and microglia (Bulters et al., 2018). After SAH, peroxynitrite can be produced by the reaction of NO with superoxide radicals, a highly oxidative species. These peroxides damage the neurons that produce NO and reduce NO production. More importantly, hemoglobin released by subarachnoid blood inhibits the activity of endothelial NO synthase (eNOS), exacerbating the decrease in NO production (Sabri et al., 2011; Lenz et al., 2021). As a result, the availability of NO to vascular smooth muscle cells is reduced, leading to vasoconstriction (Li Q. et al., 2016, Li J. et al., 2016). In addition to regulating cerebrovascular tension, NO inhibits the formation of thrombocytopenic microthrombosis (Voetsch et al., 2004). As the result of the combination of these factors, cerebral perfusion is significantly reduced leading to neuronal dysfunction (Takeuchi et al., 2006). Reduced bioavailability of NO reduces the cortical diffusion depolarization threshold (Petzold et al., 2008). This leads to diffuse ischemia and neuronal death (Terpolilli et al., 2016).

### Hemoglobin Causing Encephaledema

Many studies have shown that cerebral edema in SAH is a biphasic phenomenon (Serrone et al., 2015). The formation of early cerebral edema is a direct result of early ischemic injury during initial bleeding, while subsequent delayed edema appears to be caused by BBB dysfunction (Hayman et al., 2017). There is evidence to show that hemoglobin and its metabolites can cause brain edema. In a model of intracranial hemorrhage (ICH), the iron deposition around the hematoma gradually increases after injection of autologous blood into the brain parenchyma of rats. Meanwhile, the water content in the brain tissue around the hematoma also gradually increases (Huang et al., 2002). However, the degree of cerebral edema in rats is significantly reduced by chelating agents. Immunohistochemical analysis shows that aquaporin (AQP)4 is highly expressed in astrocytes. Therefore, iron overload and AQP4 may play a key role in the formation of hemoglobin-mediated brain edema after ICH (Qing et al., 2009). Hemoglobin-induced oxidative stress can increase expression of matrix metalloproteinase (MMP)-9 and lead to BBB dysfunction (Katsu et al., 2010; Ding et al., 2014). In view of this, delayed edema after SAH is thought to be caused at least in part by hemoglobin and its breakdown products (Urday et al., 2015).

## Immunocyte Reaction

Aside from hemoglobin activating SAH and producing ROS that damage the CNS, some inflammatory cell infiltrates cause further damage. Unlike other organs/tissues, the CNS hosts a variety of innate and peripheral immune cells. Analysis of blood, CSF and tissue sections from patients with SAH has revealed that peripheral neutrophils and monocytes/macrophages, as well as central resident microglia and astrocytes contribute most to post-SAH inflammation. However, as the course of the disease changes, so too does the immune cell spectrum involved in the inflammatory response. Recent studies have found that the accumulation and activation of neutrophils and microglia peak twice during the course of SAH, which corresponds to early and late neuronal apoptosis after SAH. However, monocytes and macrophages seem to increase from the subacute phase (Coulibaly et al., 2020). This suggests that different immune cells play different roles in the post-SAH inflammatory response. However, as the disease course changes, so too does the immune cell spectrum involved in the inflammatory response. Recent studies have found that the accumulation and activation of neutrophils and microglia peak twice during the course of SAH, which corresponds to early and late neuronal apoptosis after SAH. However, monocytes and macrophages seem to increase from the subacute phase (Coulibaly et al., 2020). This seems to suggest that different immune cells play different roles in the post-SAH inflammatory response.

### Microglia

Microglia act as the resident macrophages of the CNS and are an important component of innate and adaptive immune responses. TLR-mediated microglial activation leads to production of several inflammatory mediators that rapidly respond to different stimuli, such as DAMPs (Atangana et al., 2017). TLR4 is most abundantly expressed on microglial membranes (Lehnardt, 2010; Li et al., 2021). TLR4 and other PRRs lead to the activation of downstream inflammatory signaling cascades, including the NF-κB, MyD88/TRIF, and MAPK pathways (Geraghty et al., 2019). However, at different stages in SAH, microglia exhibit different phenotypes: the proinflammatory M1 phenotype predominates in the early stages and this is gradually replaced by the anti-inflammatory M2 phenotype as the disease progresses (Zheng et al., 2020). Between them, M1 has the ability to release proinflammatory cytokines, such as TNF-α and IL-6 (Hu et al., 2015). In animal models, increased expression of proinflammatory cytokines is associated with poor prognosis of SAH (Kooijman et al., 2014; Wang et al., 2021). Neuronal cell death and microglial cell accumulation follow similar time courses. Thus, microglial accumulation can cause secondary brain damage after SAH (Schneider et al., 2015). Therefore, early post-SAH promotion of activation of these microglia toward an anti-inflammatory phenotype may have a neuroprotective effect (Schneider et al., 2018).

### Astrocytes

Astrocytes are the most abundant glial cells in the CNS. They play an important role in maintaining the integrity of the BBB and supporting the activity of neurons (Cunningham et al., 2019). Astrocytes can differentiate into different phenotypes under different stimuli, namely proinflammatory type A1 and anti-inflammatory type A2 (Shinozaki et al., 2017; Hyvärinen et al., 2019). DAMP-mediated activation of TLR contributes to the acquisition of A1 phenotype in astrocytes (Xu et al., 2018). TLR expression is low in the astrocytes of healthy individuals. However, TLRs, especially TLR-4, are abundant on the surface of the astrocyte membrane in the event of injury or inflammation (Azam et al., 2019). After SAH, various DAMPs are released into the subarachnoid space, promoting the activation of proinflammatory phenotype through TLRs (Ghaemi et al., 2018). One study found that changes in astrocyte Ca^2+^ signaling after SAH led to a neurovascular coupling response that shifts blood vessels from a diastolic to a constrictive state, and ultimately exacerbates brain tissue damage (MacVicar and Newman, 2015; Pappas et al., 2015). At the same time, the dysfunction of glutamate uptake mediated by astrocytes may be the possible mechanism of DCI after SAH (Tao C. et al., 2020, Tao K. et al., 2020). Additionally, A1 astrocytes cause depression-like behavior and cognitive dysfunction in mice (Zhang et al., 2020). Current interventions targeting type A1 astrocytes in animal models reduce neuronal death as well as neurogenesis decline and cognitive impairment (Lu et al., 2020; Zhang et al., 2021). Therefore, therapies targeting astrocytes may help improve outcomes in patients with SAH.

### Neutrophils

Neutrophils are the most abundant type of white blood cells in peripheral blood, and they are significantly increased after SAH (Gris et al., 2019; Morga et al., 2020). Activation of astrocytes challenges the integrity of the BBB and vascular permeability is increased. Many chemokines are produced in the local inflammatory response after SAH. Under the combined action of the two, numerous neutrophils enter the subarachnoid space from the peripheral circulation (Coulibaly et al., 2020; Osuka et al., 2021). Related observational studies have found that increased neutrophil-to-lymphocyte ratio is inversely associated with prognosis in patients with SAH (Giede-Jeppe et al., 2019). Neutrophils have also been found to mediate early cortical hypoperfusion in animal models (Neulen et al., 2019). On the one hand, neutrophils can release IL-6, transforming growth factor-β1 and other inflammatory cytokines to produce a cascade reaction, which plays an important role in post-SAH inflammation (Takizawa et al., 2001). On the other hand, these neutrophils can produce peroxide by NADPH oxidase (NOX) and myeloperoxidase (MPO), causing damage to neurons and other support cells in the CNS and even apoptosis and cerebral cortex insufficiency (Chu et al., 2015; Neulen et al., 2019). Recent studies have shown that the presence of NETs released by neutrophils can transform microglia into a more proinflammatory phenotype, thereby aggravating neuroinflammation after SAH and leading to poor prognosis (Hanhai et al., 2021).

### Monocytes/Macrophages

Monocytes are innate immune cells produced mainly in the bone marrow. When they are released into the circulation, they quickly differentiate into macrophages and perform different functions. Within 24 h after SAH, there is a large number of monocytes infiltrating into the site of hemorrhage and gradually increasing (Jedrzejowska-Szypułka et al., 2010; Gris et al., 2019). Monocytes mediate cerebral vasospasm after SAH in animal models, which may be the mechanism related to DCI and DIND (Jackson et al., 2021). Once monocytes infiltrate brain tissue, they mature into macrophages and take on different morphological and biochemical characteristics (Coulibaly et al., 2020). Meanwhile, peripheral circulating macrophages are recruited by monocyte chemotactic protein-1 to enter the site of injury (Lu et al., 2009; Niwa et al., 2016). Similar to microglia, monocytes and macrophages are likely to be involved in the inflammatory response or oxidative damage after SAH and lead to poor prognosis (Kwan et al., 2019; Unda et al., 2020). Therefore, further research on its mechanism will help to develop more effective treatment regimens.

### Neurotoxicity of Immunocytes

The toxic effect of immune cells on the CNS may be the result of combined action of cytokine-mediated inflammatory response and ROS-mediated oxidative stress.

#### Proinflammatory Cytokines

After SAH, proinflammatory cytokines are initially secreted by innate immune cells that reside in the CNS (Gris et al., 2019). Appropriate inflammatory response is thought to be beneficial. Then, circulating immune cells, aided by chemokines, enter the subarachnoid space and produce a storm of inflammatory cytokines that tip the balance in the wrong direction (Schneider et al., 2012; Coulibaly et al., 2020). Proinflammatory cytokines can induce brain injury by triggering apoptotic pathways, interfering with the balance of endogenous vasodilators and vasoconstrictors, and activating coagulation factors, leading to microthrombosis (Becher et al., 2017).

Currently, several factors have been identified to induce or aggravate cerebral vasoconstriction after SAH, including interleukins, TNF-α, lymphocyte function-associated antigen-1 (LFA-1), leukotrienes, arachidonic acid, von Willebrand factor, MMP-9 and vascular endothelial growth factor (Schneider et al., 2018). Typically, IL-1, IL-6, IL-8, and TNF-α reported to be correlated with the prognosis of patients with SAH (López-Cortés et al., 2000; Zeiler et al., 2017). IL-6, for example, is considered to be a biomarker for DCI-associated infarction after SAH (Ridwan et al., 2021). IL-6 regulates the expression of many genes related to inflammation, oxidative stress, and apoptosis. IL-6 mediates the endothelial barrier, resulting in dysregulation of cell connections and damage to the BBB (Blecharz-Lang et al., 2018). At the same time, vascular cell adhesion molecule-1 on vascular endothelial cells is upregulated to induce proliferation of circulating immune cells and infiltration into the CNS (Erta et al., 2012). IL-6 can induce the accumulation of abnormal proteins and molecules in neurons, leading to neurodegeneration (Kaur et al., 2019). IL-6 may also play a neuroprotective role. For example, against *N*-methyl-D-aspartic acid receptor-mediated brain excitatory toxicity (Ali et al., 2000).

The dual role of cytokines in secondary inflammation after SAH has been demonstrated. Moderate levels of cytokines contribute to injury repair; however, prolonged chronic stimulation can be harmful. Therefore, early anti-inflammatory treatment in patients with SAH will help improve prognosis.

#### Oxidative Damage

When cell are in homeostasis, there is a balance between ROS produced by mitochondria and peroxidases [such as NOX, NOS, MPO, and cyclo-oxygenase (COX)] that produce ROS and antioxidant enzymes (such as catalase, superoxide dismutase (SOD), and glutathione reductase) and endogenous antioxidant molecules (such as glutathione, ascorbic acid, and tocopherol) (Eastman et al., 2020). However, after SAH, this balance is broken and oxidative stress occurs.

##### Mitochondrial dysfunction

**FIGURE 3:**
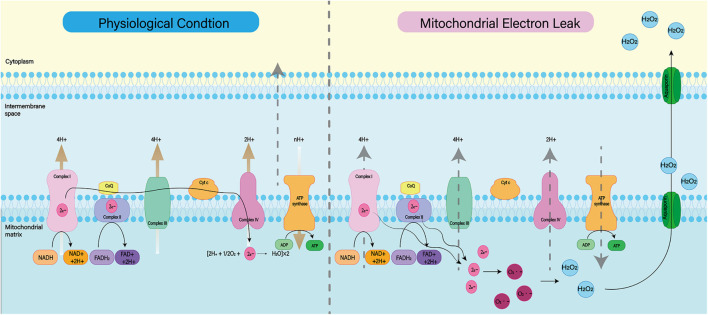
Under normal physiological conditions, mitochondrial oxidative respiratory chain composed of complex I–IV can transfer electrons and H^+^ and produce ATP together with ATP synthase. When complex I–IV is dysfunctional, electron leakage occurs. This leads to the production of ROS and H_2_O_2_.

Mitochondria are known to play a major role in tissue oxidative damage and dysfunction due to their ability to produce free radicals (Venditti and Di Meo, 2020). With the development of SAH, nerve cells in the responsible vascular supply region suffer ischemia injury, leading to mitochondrial dysfunction (Qu et al., 2016; Halcrow et al., 2021). Mitochondrial dysfunction can lead to a series of harmful consequences, including breakdown of transmembrane potential in mitochondria, disruption of mitochondrial biosynthesis, excessive production of ROS, outflow of matrix calcium, and release of apoptotic proteins (Chan, 2006). Respiratory chain complex I–IV and oxidants may be the main reasons for the enhanced mitochondrial ROS production after SAH (Figure 3; Dröse and Brandt, 2012). A growing body of evidence supports the role of mitochondrial oxidants in ROS production after intracerebral hemorrhage (Wu et al., 2019; Nolfi-Donegan et al., 2020). In addition, mitochondrial dysfunction produces ROS that contribute to CNS dysfunction (Marzatico et al., 1990). That has been proved in most neurodegenerative diseases, including Parkinson’s disease, Alzheimer’s disease, Huntington’s disease, and amyotrophic lateral sclerosis (Wang et al., 2019; Johnson et al., 2021). For SAH patients, mitochondrial dysfunction is also thought to be a key mechanism for cognitive dysfunction and poor prognosis (Youn et al., 2020). Therefore, we can reduce the nerve damage after SAH by inhibiting mitochondrial ROS production.

##### NADPH oxidase

**FIGURE 4:**
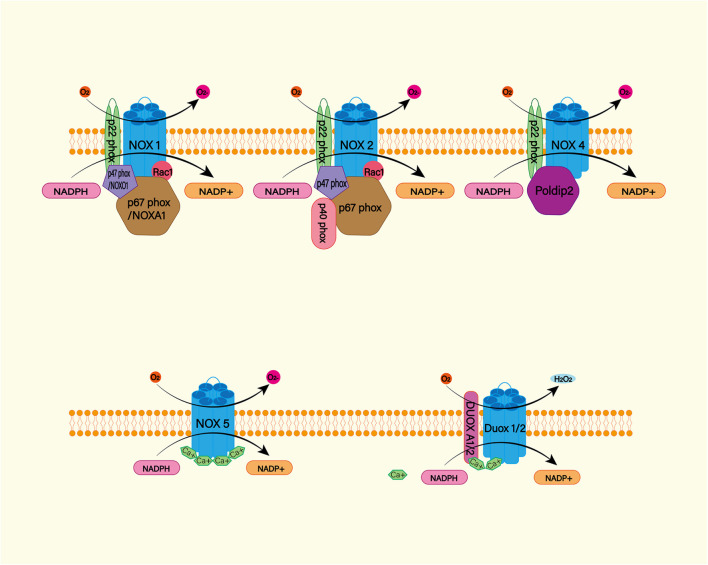
The NADPH oxidase (NOX) family consists of seven catalytic subunits (NOX1-5 and DUOX1-2), regulatory subunits p22Phox, P47Phox or Noxo1, P67phox or Noxa1, and P40phox and Rac. They are widely expressed in endothelial cells (EC), vascular smooth muscle cells (VSMC), macrophages and other cells. Specifically, NOX1, 2, 4, and 5 are highly expressed in cardiovascular tissues. NOX – mediated ROS production mainly occurs on catalytic subunit Nox or Duox. For Nox1 and Nox2, ROS production requires complex interactions of regulatory subunits in the cytoplasm. Nox4, on the other hand, requires protein termed δ-interacting protein 2 (Poldip2). In addition, the increase in intracellular calcium was sufficient to promote the activation of NOX5 and DUOX1-2.

The NADPH oxidase (NOX) family (NOX1–5 and dual oxidase (DUOX1 and 2) is an important source of ROS (Figure 4; Mittal et al., 2014). Under physiological conditions, ROS produced by NOX function as a defense mechanism against pathogens and signaling molecules. In pathological conditions, ROS cause oxidative damage through oxidative stress (Sorce et al., 2012). NOX catalyzes the transfer of two electrons through the biofilm to produce superoxide anion O2^–^^∙^ by using intracellular NADPH as electron donor and extracellular molecular oxygen as receptor. Then O2^–^^∙^ is progressively metabolized to H_2_O_2_ and ^∙^OH (Nayernia et al., 2014). NOX is expressed widely in CNS cells, while oligodendrocytes may be the only CNS cells that do not express NOX (Cinelli et al., 2020). NOX2 and 4 appear to be the major subtypes expressed in the brain under physiological conditions, with NOX2 being the most abundant (Miller et al., 2006). A study found significantly increased levels of NOX2 and 4 proteins in perihematoma neurons and astrocytes in SAH patients (Zhang et al., 2017). In animal models, NOX can trigger delayed cerebral vasospasm after SAH (Kim et al., 2002). NOX is associated with neurodegenerative diseases such as Alzheimer’s disease (Ganguly et al., 2021). This suggests that NOX is likely to be a risk factor for DIND after SAH. Therefore, NOX is a potential target for the treatment and prognosis improvement of SAH.

##### Myeloperoxidase

**FIGURE 5:**
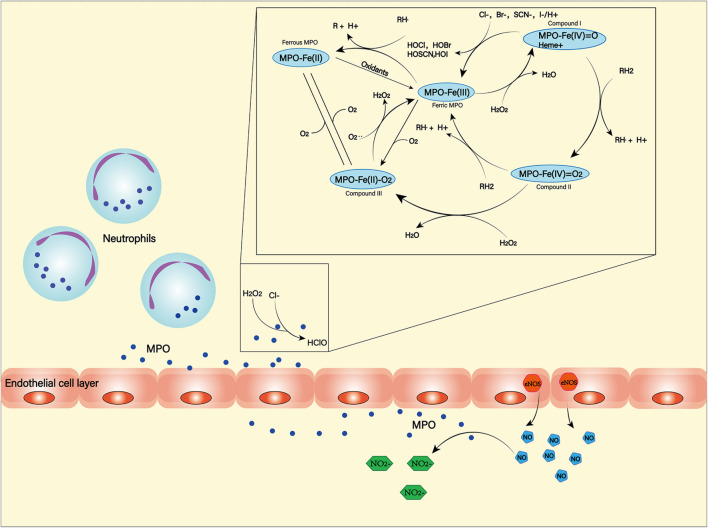
Myeloperoxidase (MPO) is produced and secreted mainly by neutrophils. It can produce a variety of oxidation products through halogenation cycle and peroxidation cycle and then cause damage to tissues and cells. In halogen cycle, MPO catalyzes halogen to produce HOCl and other strong oxides. In the peroxidation cycle, MPO can react with oxidizable molecules (RH) to form free radical intermediates. In addition, MPO can react with NO generated by NOS to form NO2^–^.

Myeloperoxidase (MPO) is a heme-containing peroxidase. It was found in the primary azurophilic granules of neutrophils and a small amount in the primary lysosomes of monocytes (Guilpain et al., 2008; Aratani, 2018). Neutrophils, in the middle and late stages of SAH, are recruited by chemokines into the subarachnoid space. While producing ROS through NOX, they can also use MPO to produce ROS (Winterbourn et al., 2016). Hypochlorous acid is the main product of MPO (Kargapolova et al., 2021), which can damage lipids, proteins and DNA due to its high dispersibility and oxidative activity (Figure 5; Chen J. et al., 2020). MPO-mediated nerve damage has been shown to cause cognitive impairment and neurodegeneration (Ray and Katyal, 2016). In SAH patients, serum MPO levels are positively correlated with DCI (Lim et al., 2012), and this has been confirmed in animal models (Oruckaptan et al., 2000). These suggest a negative role of MPO in the course of SAH.

##### NOS

**FIGURE 6:**
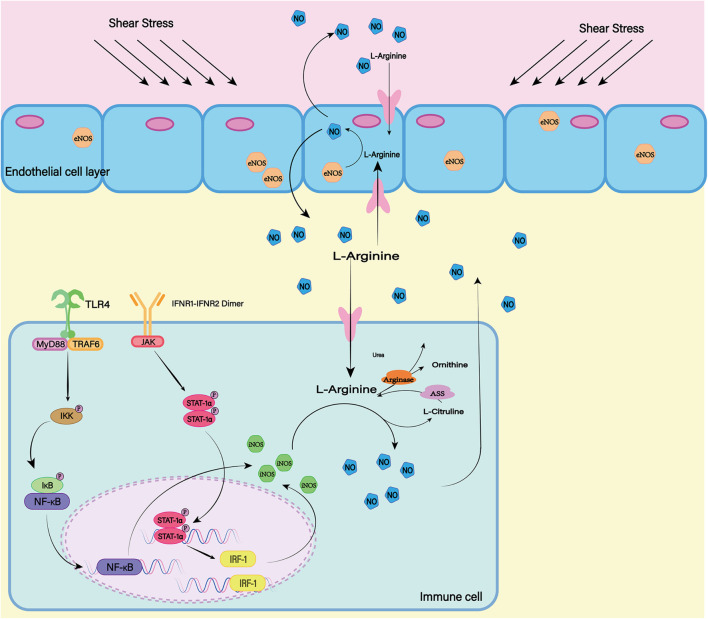
In mammals, there are three isotypes of nitric oxide synthase (NOS) (eNOS, iNOS, and nNOS). ENOS is the most important source of NO in endothelial cells. The shear stress of blood flow on the vascular wall is the main mechanism by which eNOS produces NO. INOS can be induced in a variety of cell types. LPS as a proinflammatory medium can induce the expression of iNOS. At the same time, the transcription factors NF-κB and STAT-1α are believed to be necessary for iNOS transcription in most cells. nNOS is highly expressed mainly in peripheral nerve fibers and is thought to have a protective effect on atherosclerosis. The release of L-Glutamate (L-Glu) from baroreceptors activates nNOS and promotes the production of NO. Although the expression mechanisms of the three isotypes are different, all of them can generate NO and L-Citrulline (L-CCP) as the substrate.

The nitric oxide synthase (NOS) family consists of three subtypes: endothelial NOS (eNOS), neuronal NOS (nNOS), and inductive NOS (iNOS) (Figure 6). The first two are constitutively expressed, while the latter is usually induced during inflammation (Pluta, 2006). In most cell types, expression of iNOS requires stimulation by cytokines or other inflammatory products. As the resident innate immune cells in the CNS, microglial cells are among the earliest activated immune cells after SAH (Coulibaly and Provencio, 2020). After SAH, microglia can transform into M1 phenotype, which can express iNOS depending on transcription factors including hypoxia-inducible factor-1 and NF-κB responding to inflammatory factors and hypoxic environment (Robinson et al., 2011; Rawlinson et al., 2020). Subsequently iNOS can increases NO levels and lead to free-radical-mediated neuronal damage (Bai et al., 2020).

##### COX

COX, also known as prostaglandin oxidase reductase, is a bifunctional enzyme with activities of cyclo-oxygenase and catalase. It has two isozymes, COX-1 and COX-2, and is the key enzyme to catalyze the conversion of arachidonic acid to prostaglandins (Rouzer and Marnett, 2020). Thromboxane A2 (TXA2) synthesized by COX-1 induces platelet aggregation, vasoconstriction, and smooth muscle proliferation, while prostacyclin synthesized by COX-2 in the vascular endothelium antagonizes TXA2 in the macrovascular endothelium through smooth muscle relaxation and vasodilation (Young et al., 2012). In animal models, COX-2 overexpression is found in arterial endothelial cells after SAH, while COX-1 expression level does not change significantly (Tran Dinh et al., 2001). Subsequent studies have shown that COX-2 inhibitors are effective in preventing brain edema and protecting neurological function (Ayer et al., 2011). However, nonsteroidal anti-inflammatory drugs (NSAIDs), such as aspirin, may also increase the risk of rebleeding while reducing SAH inflammation (Roumie et al., 2008; Parkhutik et al., 2012). Therefore, the use of COX inhibitors in the treatment of SAH should be carefully considered.

## Endothelial Cell Dysfunction

Blood-brain barrier is mainly composed of tightly connected vascular endothelial cells, astrocyte terminal, and extracellular matrix. Among them, normal endothelial cells play an important role in maintaining BBB, regulating thrombosis and regulating vascular tone. After SAH, the integrity of the BBB is destroyed (Peeyush Kumar et al., 2019). Therefore, vascular endothelial damage will damage the blood-brain barrier, exacerbating neurological dysfunction (Graves and Baker, 2020).

After SAH, the CNS experiences a transient systemic ischemia. Normally, vasoactive substances have a delicate balance between vasodilation and contraction. Early in the course of the disease, the balance becomes out of balance, with endothelial cells responding more to vasoconstrictors and less to vasodilators (Geraghty et al., 2019; Lenz et al., 2021). Therefore, the cerebrovascular contraction and even spasm are stimulated by a variety of vasoconstrictor substances such as endothelin. In quick succession, there will be lack of cerebral blood flow and cerebral perfusion (Jullienne et al., 2016). In the ischemic environment, in addition to impaired nerve cell function, vascular endothelial cells undergo morphological changes and become dysfunctional (Sehba and Friedrich, 2013; Østergaard et al., 2013).

As the disease progresses, secondary inflammation and oxidative stress further damage the endothelial cells. Under the joint action of many factors, endothelial cell dysfunction is aggravated and eventually apoptosis. In addition, damaged endothelial cells release substances such as matrix metalloproteinase 9 (MMP-9) (Wang et al., 2018). Mmp-9 can degrade the extracellular vascular matrix, including collagen IV, laminin, and fibronectin, etc. (Lu et al., 2011). Therefore, the integrity of the BBB is lost. This exposes nerve cells to a large amount of harmful substances and causes brain tissue edema and other central nervous dysfunction (Willis et al., 2008; Hayman et al., 2017; Swissa et al., 2019).

## Hemoglobin as a Therapeutic Target

Hemoglobin causes direct and indirect neurotoxicity; therefore, therapies that targeting hemoglobin are important for patients with SAH.

### Physical Clearance

Surgery is the only way to completely remove the blood clot. Especially for SAH patients with large hematomas or ruptures in the cerebroventricular system, surgery can effectively remove the hematoma and reduce the occurrence of vasospasm (Zhang et al., 2001). However, due to the anatomical structure of SAH, blood can be widely distributed in various parts of the subarachnoid space. Therefore, for patients with small hematomas and mild symptoms, surgical treatment is no longer appropriate.

For patients in whom craniotomy for removal of hematoma is not recommended, continuous drainage of CSF by external ventricular drainage and lumbar cistern drainage can be performed to reduce the hemoglobin concentration in CSF (Wolf, 2015). Continuous CSF drainage can significantly reduce the incidence of vasospasm (Klimo et al., 2004; Alcalá-Cerra et al., 2016). However, CSF drainage is of limited use and it does not clear the blood clot that has formed. Blood clots can still release hemoglobin, which can damage brain tissue. Therefore, CSF drainage results in a small improvement in long-term outcomes after SAH (Al-Tamimi et al., 2012).

In recent years, CSF drainage combined with intrathecal drug therapy has received attention. A randomized controlled trial in Japan used magnesium sulfate solution for intrathecal irrigation. Although vasospasm was significantly improved, the incidence of DCI and functional outcomes in patients with SAH were not significantly improved (Yamamoto et al., 2016). The combined intrathecal use of thrombolytic agents has not yielded satisfactory results (Etminan et al., 2013). It has also been found that intrathecal use of thrombolytic agents increases inflammation. However, this may be due to the release of hematoma breakdown products rather than thrombolytic drugs (Kramer et al., 2015).

### Enhancing Endogenous Hemoglobin Clearance

**FIGURE 7:**
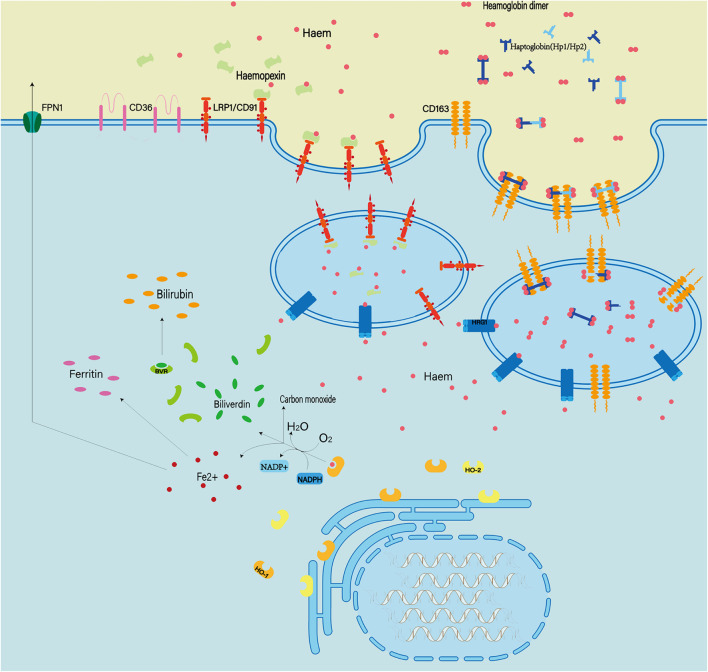
Extracellular hemoglobin can be metabolized into hemoglobin dimer as well as heme. The hemoglobin dimer can bind to the haptoglobin and enter the cell mediated by CD163. Soon afterward, the hemoglobin dimer that enters the cell will be decomposed into heme. On the other hand, extracellular heme can bind to hemopexin and enter the cell mediated by CD91. In quick succession, heme and oxygen in the cell are catalyzed by heme oxygenased (HO1/2) to produce Fe^2+^ ions, CO and bilirubin. In addition, abnormal red blood cells can also be cleared by phagocytosis mediated by the expression of type II scavenger receptor (CD36) on the phagocytic membrane.

Hemoglobin can be engulfed by cells in three ways: erythrophagocytosis, endocytosis of erythrocytes mediated by haptoglobin, and endocytosis of heme mediated by hemopexin (Figure 7; Bulters et al., 2018; Pan et al., 2020). These mechanisms of endogenous hemoglobin clearance provide an entry point for therapeutic regimens that target hemoglobin.

#### Erythrophagocytosis

Erythrophagocytosis is the removal of abnormal RBCs by monocytes/macrophages with type II scavenger receptors (CD36) expressed on their membranes. After SAH, macrophages/microglia recognize exposure to phosphatidylserine via CD36 and mediate erythrophagocytosis. In particular, transcription factor Nrf2 (nuclear factor- erythroid 2 p45-related factor 2) regulates the expression of CD36 on microglia, thereby improving RBC clearance (Zhao et al., 2015). Recent studies have shown that CD47 can enhance RBC clearance (Tao K. et al., 2020). However, the exact role of CD47 in erythrocyte clearance remains to be further studied. Although erythrophagocytosis appears to be an effective scavenging mechanism, macrophages that ingest more than two erythrocytes undergo cell death and release heme and iron into the extracellular matrix (Knutson et al., 2005). This means that the use of erythrophagocytosis does not provide beneficial help to clear RBCs.

#### Haptoglobin

Haptoglobin, an acute-phase reactant produced primarily by the liver, is the primary hemoglobin-binding protein in humans and most mammals (Buehler et al., 2020). The hemoglobin dimer produced after RBC rupture can immediately and irreversibly bind to binding haptoglobin, which is known as one of the strongest naturally occurring noncovalent interactions (Andersen et al., 2017). The binding of haptoglobin to hemoglobin reduces the redox potential of hemoglobin to reduce oxidative damage and prevent the release of heme and the generation of free iron during degradation of heme (Garland et al., 2020). Following the formation of haptoglobin–hemoglobin complex, CD163 (the scavenger membrane receptor) on macrophages and microglia can internalize the complex, leading to decomposition of hemoglobin in phagolysosomes (Jing et al., 2018).

The researchers found that haptoglobin has three distinct genotypes, homozygous HP1–HP1 and HP2–HP2, and heterozygous HP2–HP1, due to the two major alleles of the haptoglobin gene presented on chromosome 16 (Nielsen and Moestrup, 2009). HP1–HP1 has the best affinity with hemoglobin, and hemoglobin combined with HP1–HP1 is cleared fastest (Azarov et al., 2008; Morton et al., 2020). In the 1980s, Japanese scientists were using haptoglobin to treat hemolysis secondary to burns (Imaizumi et al., 1994). Since then, scientists have tried using haptoglobin in animal models with some success (Lipiski et al., 2013; Schaer et al., 2018). However, haptoglobin therapies have not been reported in clinical trials. In the future, many advances in recombinant protein design, truncated binding constructs, and fusion protein design will contribute to novel haptoglobin-based therapies.

#### Hemopexin

Hemopexin is an acute phase plasma glycoprotein that is primarily produced by the liver and released into plasma, but it can also be expressed by neurons and glia (Morris et al., 1993; Tolosano et al., 2010). However, the level of hemopexin in the CSF is usually 10 times lower than the normal circulating level (Garland et al., 2016). Hemopexin can bind heme with high affinity to form complexes. Subsequently, the complex can bind to CD91 (low-density lipoprotein receptor-related protein-1). CD91 is a transmembrane protein that is expressed on membranes of various cells (such as macrophages, astrocytes, and oligodendrocytes) and mediates endocytosis of the complexes, leading to clearance of heme. However, one study found that approximately one-third of patients with SAH had elevated heme-binding proteins in the CSF. Meanwhile, these patients were more likely to develop DCI and had poorer neurological outcomes (Garland et al., 2016). Animal studies have shown that hemopexin reduces early post-ICH damage, but does not reduce neurological deficits, inflammatory cell infiltration, or perihematoma cell viability and improve patients prognosis in the long term (Chen-Roetling et al., 2021). Nevertheless, hemopexin is recognized as protective after systemic hemolysis and may be helpful for patients with SAH (Smith and McCulloh, 2015). However, further study is still needed to confirm the neuroprotective effects of hemopexin after SAH (Griffiths et al., 2020). In the future, research should focus on more effective targeting of CNS delivery and the sustainability of efficacy.

## Anti-Inflammatory Therapy

Clinical trials of anti-inflammatory drugs have only been studied in a small number of subjects. Therapies with steroids, statins and NSAIDs have yet to show significant clinical benefit (de Oliveira Manoel and Macdonald, 2018).

### Steroid Hormones

Steroid hormones are effective in combating inflammation and inhibiting the production of proinflammatory cytokines (Whitehouse, 2011). A retrospective study found that dexamethasone treatment appeared to reduce the risk of adverse outcomes after SAH (Czorlich et al., 2017). However, another study showed that dexamethasone use reduced poor outcomes, but not for DCI (Mohney et al., 2018). This may be because steroid hormones help maintain the water and electrolyte balance and reduce brain edema (Mistry et al., 2016).

### Statins

Statins, or 3-hydroxy-3-methylglutaryl-CoA inhibitors, can reduce total cholesterol and low-density lipoprotein (LDL), as well as triglyceride. They also increase high-density lipoprotein. Simvastatin has been shown to reduce the occurrence of DCI in animal models (Sugawara et al., 2008). This may be because statins exert a neuroprotective effect through a cholesterol-dependent mechanism (Tseng et al., 2007). However, a retrospective trial found that atorvastatin reduced cerebral vasospasm and infarction after SAH, but had little effect on long-term prognosis (Chen S. et al., 2020). Therefore, more evidence is needed to support the need for statins in patients with SAH (Bohara et al., 2021).

### COX Inhibitors

As mentioned above, COX is important in the pathophysiological process of SAH. Aspirin, as a nonselective COX inhibitor, inhibits the synthesis of TXA2 and prostacyclin simultaneously. Thus, aspirin inhibits the formation of microthrombosis and reduces the inflammatory response in SAH (Muroi et al., 2014; Darkwah Oppong et al., 2019). However, there are considerable differences of opinion regarding the use of NSAIDs. Although feasible in theory, they seem to have little effect in clinical practice (Young et al., 2012).

### Other Potentially Anti-inflammatory Drugs

Minocycline, a tetracycline antibiotic, has recently been found to prevent inflammation and p53-related apoptosis induced by NLRP3 inflammasomes (Li Q. et al., 2016, Li J. et al., 2016). In addition, fluoxetine, which has been shown to have anti-inflammatory effects in many diseases, attenuates NLRP3 inflammasome and caspase-1 activation through autophagy activation in animal models (Li et al., 2017). Melatonin has also been found to reduce the inflammatory response and be beneficial for early post-SAH brain injury (Dong et al., 2016).

## Antioxidant Therapy

Excessive ROS can cause irreversible oxidative damage to cells, proteins, lipids, and DNA, leading to cell necrosis or apoptosis and subsequent cell or tissue damage. At present, there are two possible therapeutic approaches to reduce ROS: enhancing the activity of endogenous antioxidant enzymes, and preventing or reducing the production of peroxides.

### Enhance Antioxidant Capacity: Nrf2 Regulation

**FIGURE 8:**
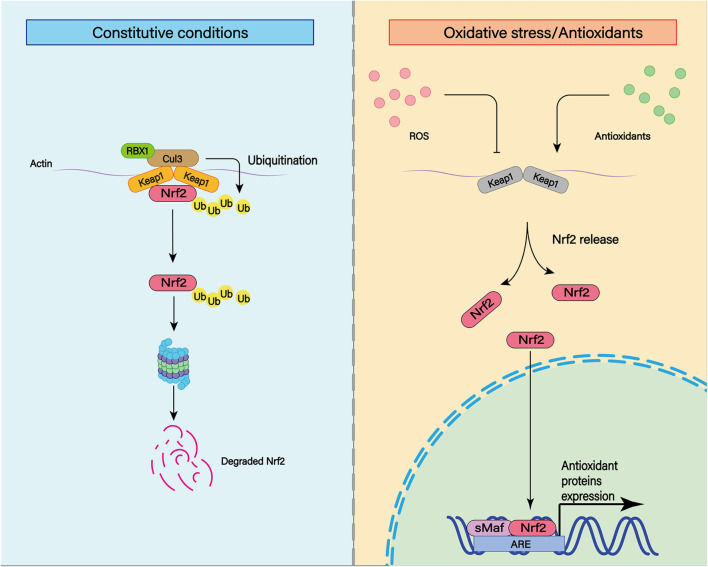
The transcription factor nuclear factor erythroid-derived 2-like 2 (Nrf2) plays an important role in cellular antioxidant and other important physiological processes. Under physiological conditions, Nrf2 can be ubiquitinated and bind to Keap1. Subsequently, Nrf2 can be degraded by keAP1-dependent proteasome. In addition to ubiquitination, various post-translational modifications such as phosphorylation can affect the stability of Nrf2 structure. Under moderate oxidative stress and antioxidant stimulation, Nrf2 can enhance its stability through various post-translational modifications such as phosphorylation. At the same time, Nrf2 with enhanced stability can be translocated to the nucleus and combined with the cis-acting element ARE to activate the transcription of antioxidant genes.

Nuclear factor-erythroid 2 p45-related factor 2 is a transcription factor that recognizes antioxidant response elements (AREs) to regulate the expression of a variety of genes (Figure 8; Shao et al., 2020). Nrf2 binds to the Kelch-like ECH-associated protein 1 (Keap1) in the cytoplasm through its binding domain. Therefore, the function of Nrf2 is regulated by Keap1 (Itoh et al., 1999). The Nrf2 system is widely expressed in CNS and is usually upregulated in response to inflammation and brain injury (Sandberg et al., 2014).

In animal experiments, Nrf2 expression is upregulated in cerebral arteries of rats after experimental SAH (Wang et al., 2010). It attenuates early brain injury such as cerebral edema, BBB injury and cortical cell apoptosis through the Nrf2–ARE pathway (Chen et al., 2011). Nrf2 can also reduce the occurrence of cerebral vasospasm (Zhao et al., 2016). After the Nrf2 gene is knocked out, brain injury of SAH rats is aggravated, including increased cerebral edema, BBB destruction, apoptosis of nerve cells, and severe neurological impairment (Li et al., 2014). Thus, existing studies have demonstrated that Nrf2 plays an important role in alleviating secondary complications induced by SAH.

Currently, sulforaphane, curcumin, astaxanthin, lycopene, melatonin, erythropoietin, and other Nrf2 system activators are available. All of this works by binding to Keap1 to release Nrf2. Released Nrf2 translocation into the nucleus leads to increased transcription (Zolnourian et al., 2019). Currently, there is growing clinical interest in the use of Nrf2 activators in the treatment of SAH (Guo et al., 2018; Zolnourian et al., 2020). We may use this mechanism to treat patients with SAH in the future.

### Reducing Peroxides

**FIGURE 9:**
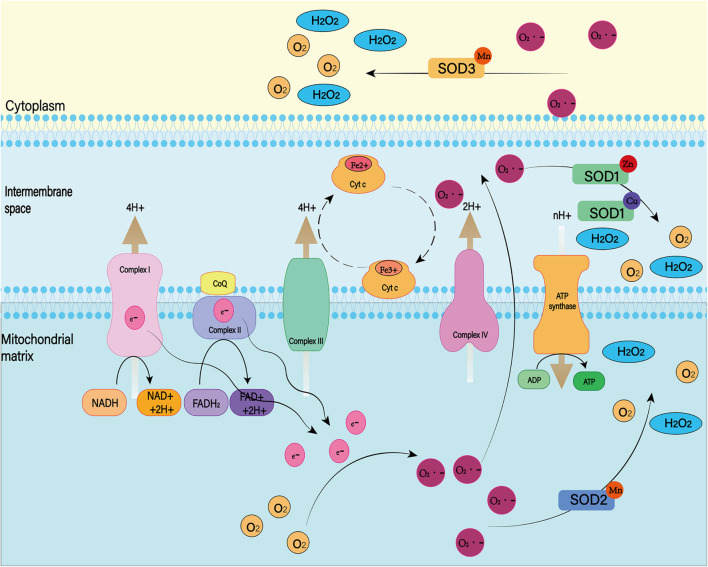
There are many isotypes of SOD. Among them, SOD1, SOD2, and SOD3 play major roles in the cell. Both were able to reduce superoxide to produce H_2_O_2_ and O_2_, but they worked in different places. SOD1 exists in the mitochondrial membrane space, SOD2 is distributed in the mitochondrial matrix, and SOD3 is distributed in the cell matrix.

**FIGURE 10:**
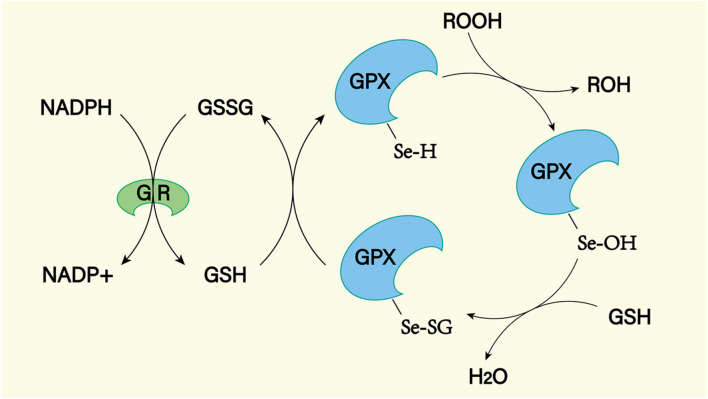
The GPX mediated reduction of hydrogen peroxide and lipid hydroperoxides involves the formation of multiple intermediates with the assistance of glutathione (GSH). The selenol (–SeH) of GPX reacts with peroxides to form selenic acid (Se-OH). Then the selenic acid is reduced by GSH to form the Se-SG intermediate of GPX. GPX-Se-SG was reduced by the second GSH to form GSSG. GSSG can be reduced by glutathione reductase (GR) as reduced equivalent by NADPH.

Superoxide dismutase, glutathione peroxidase (GPX), and catalase are important peroxidase scavengers in the CNS (Figures 9, 10; Lewén et al., 2000). However, the antioxidant capacity of these enzyme systems is reduced after SAH (Marzatico et al., 1993). This leads to a negative effect on the antioxidant stress after SAH. For example, decreased concentrations of SOD in plasma and CSF have been shown to be associated with long-term poor outcomes after SAH (Krenzlin et al., 2021). In addition, gene transfer of SOD was found to reduce cerebral vasospasm after experimental SAH (Watanabe et al., 2003). So far, no clinical treatment associated with exogenous antioxidant enzymes has been reported. In the future, exogenous supplementation of these enzymes will possibly reduce the level of oxidative stress in SAH patients, reduce the damage of peroxides to the CNS, and improve prognosis.

## Conclusion

Subarachnoid hemorrhage is a complex disease with multiple mechanisms involved in its pathophysiology. Many studies have shown that inflammatory response and oxidative stress play an important role in the progression and prognosis of SAH. It has been demonstrated that inflammatory response and oxidative stress have adverse effects on CNS function. They play a negative role in the pathophysiological processes of cognitive dysfunction, neurodegeneration and psychiatric diseases.

RBCs are the earliest cell component to enter the subarachnoid space after SAH and become the initiator of secondary SAH inflammation. Subsequently, microglia and immune cells such as neutrophils recruited from the peripheral circulation are the main bearers of SAH inflammatory response, producing and releasing a large number of proinflammatory cytokines and ROS. These, together with ROS formed during hemoglobin metabolism, which is released after rupture of RBCs, mediate damage to the CNS.

Treatment regimens (surgery and drugs) targeting secondary inflammation and oxidative stress after SAH have been shown to improve the outcomes of patients with SAH. However, there is not enough detailed basic research and sufficient, well-controlled clinical trials to draw definitive conclusions about safety and efficacy. In the future, more effective treatment regimens will be developed to help prevent complications and improve outcomes.

## Author Contributions

FW: conceptualization, writing – original draft, and drawing-graph. ZL: conceptualization and drawing-graph. GL and LZ: writing – original draft. KH and ZW: drawing-graph. JS and RZ: writing – review and editing. All authors contributed to the article and approved the submitted version.

## Conflict of Interest

The authors declare that the research was conducted in the absence of any commercial or financial relationships that could be construed as a potential conflict of interest.

## Publisher’s Note

All claims expressed in this article are solely those of the authors and do not necessarily represent those of their affiliated organizations, or those of the publisher, the editors and the reviewers. Any product that may be evaluated in this article, or claim that may be made by its manufacturer, is not guaranteed or endorsed by the publisher.
